# Ribosomal Protein S27/Metallopanstimulin-1 (RPS27) in Glioma—A New Disease Biomarker?

**DOI:** 10.3390/cancers12051085

**Published:** 2020-04-27

**Authors:** Jonas Feldheim, Almuth F. Kessler, Dominik Schmitt, Ellaine Salvador, Camelia M. Monoranu, Julia J. Feldheim, Ralf-Ingo Ernestus, Mario Löhr, Carsten Hagemann

**Affiliations:** 1 Tumorbiology Laboratory, Department of Neurosurgery, University of Würzburg, Josef-Schneider-Str. 11, D-97080 Würzburg, Germany; jonas.feldheim@gmail.com (J.F.); Kessler_A1@ukw.de (A.F.K.); Schmitt_D8@ukw.de (D.S.); Salvador_E@ukw.de (E.S.); Feldheim.Julia@gmail.com (J.J.F.); Ernestus_R@ukw.de (R.-I.E.); Loehr_M1@ukw.de (M.L.); 2 Department of Neuropathology, Institute of Pathology, University of Würzburg, Josef-Schneider-Str. 2, D-97080 Würzburg, Germany; camelia-maria.monoranu@mail.uni-wuerzburg.de

**Keywords:** glioblastoma multiforme, low-grade glioma, astrocytoma, recurrence, relapse, mRNA, protein, brain, expression, MPS1

## Abstract

Despite its significant overexpression in several malignant neoplasms, the expression of RPS27 in the central nervous system (CNS) is widely unknown. We identified the cell types expressing RPS27 in the CNS under normal and disease conditions. We acquired specimens of healthy brain (NB), adult pilocytic astrocytoma (PA) World Health Organization (WHO) grade I, anaplastic PA WHO grade III, gliomas WHO grade II/III with or without isocitrate dehydrogenase (IDH) mutation, and glioblastoma multiforme (GBM). RPS27 protein expression was examined by immunohistochemistry and double-fluorescence staining and its mRNA expression quantified by RT-PCR. Patients’ clinical and tumor characteristics were collected retrospectively. RPS27 protein was specifically expressed in tumor cells and neurons, but not in healthy astrocytes. In tumor tissue, most macrophages were positive, while this was rarely the case in inflamed tissue. Compared to NB, RPS27 mRNA was in mean 6.2- and 8.8-fold enhanced in gliomas WHO grade II/III with (*p* < 0.01) and without IDH mutation (*p* = 0.01), respectively. GBM displayed a 4.6-fold increased mean expression (*p* = 0.02). Although RPS27 expression levels did not affect the patients’ survival, their association with tumor cells and tumor-associated macrophages provides a rationale for a future investigation of a potential function during gliomagenesis and tumor immune response.

## 1. Introduction

In 2016, the World Health Organization (WHO) published a new classification for tumors of the central nervous system (CNS) that signaled a major paradigm shift [1]. While brain tumors were previously mainly judged by their histological appearance [2], the significance of molecular markers increased in the recent classification [1]. Isocitrate dehydrogenase (IDH) mutant gliomas WHO grade II/III (IDHmut glioma), for instance, mainly arise in younger patients [3] and are associated with a comparably good prognosis [4]. In contrast, the growth and prognosis of IDH-wildtype astrocytomas (IDHwt glioma) tend towards that of glioblastoma multiforme (GBM). As a matter of fact, they might not even be an independent group, but mainly constitute misdiagnosed or early detected GBM [5,6].

GBM is the most common primary tumor of the CNS. It still represents a major clinical challenge. Even with the current standard therapy, which encompasses surgical resection, radiation, and temozolomide (TMZ)-based chemotherapy, the prognosis remains rather poor [7,8]. Although the introduction of tumor treating fields (TTFields) to the therapeutic scheme and, most recently, the results of the CeTeG/NOA-09 trial, which proved a benefit of combined TMZ and lomustine chemotherapy for O6-methylguanine-DNA methyltransferase (MGMT)-methylated patients, promise hope for improving perspectives, identifying new therapeutic targets and approaches is still a major task in GBM research [9,10,11,12,13].

Recurrence tends to be the rule rather than the exception in GBM, creating additional obstacles as relapses often gain in aggressiveness and further resistance to therapy [14]. Furthermore, the efficacy of some therapeutics depends on specific molecular characteristics. For instance, the alkylating chemotherapeutic TMZ is most effective in tumors with methylated O6-methylguanine-DNA methyltransferase (MGMT) gene promoter [8,15,16,17]. MGMT is a DNA repair enzyme removing alkyl groups from guanine in the DNA, thereby counteracting TMZ-induced DNA damage [3,18]. Therefore, the treatment benefit is restricted for some patients with an unfavorable molecular profile, making the identification of new glioma-subgroup independent molecular markers or therapeutic targets highly desirable.

Ribosomal-protein S27 (RPS27), also known as Metallopanstimulin-1 (MPS1), is part of the human ribosome 40S subunit and mainly located in the cytoplasm but can also be found in the cell’s nucleus. It can interact with DNA via its C4-type zinc finger and might prove a role in the initiation of translation as it is covalently linked to the eukaryotic initiation factor 3 [19].

RPS27 is overexpressed in various proliferating tissues, as well as several human malignancies, such as prostate, liver, stomach, colon, and head and neck cancer [20,21,22,23,24,25]. Interestingly, overexpression in gastric cancer did not only correlate with tumor-node-metastasis and later stages of malignant transformation, but the silencing of this overexpression inhibited cell growth and induced apoptosis in vitro and in vivo [25]. In addition, it can be measured in the serum of patients with different cancers and therefore has been suggested as a liquid biopsy tumor marker [26].

Despite its significant elevation in patients with a multitude of different oncological diseases [26], the role of RPS27 in the CNS is widely unknown. Fernandez-Pol [27] detected its expression in Purkinje cells of the cerebellum and also reported an intermediate expression of RPS27 in the GBM cell line U138 [28]. Therefore, we aimed to (1) identify the RPS27 expressing cell types in the normal CNS, inflammatory and neurodegenerative brain diseases, as well as in glial tumors and (2) examine associations between the RPS27 mRNA expression and tumor/patient characteristics, such as the molecular profile, treatment, and outcome.

## 2. Results

### 2.1. Patient Cohort

We collected specimen of healthy brain (NB) (epilepsy surgery n = 5, autopsy *n* = 3), Alzheimer’s disease (cerebrum and cerebellum *n* = 2), multiple sclerosis (cerebrum and cerebellum *n* = 1, cerebrum only *n* = 2), encephalitis (cerebrum *n* = 2), adult pilocytic astrocytoma (PA) WHO grade I (*n* = 4), anaplastic PA WHO grade III (APA *n* = 5), gliomas WHO grade II/III (isocitrate dehydrogenase (IDH)-mutated tumors with the histological appearance of WHO grade II and III gliomas—IDHmut glioma *n* = 28; IDH wildtype tumors with the histological appearance of WHO grade II and III gliomas—IDHwt glioma *n* = 13), and GBM with different growth patterns at first diagnosis and relapse (*n* = 76). We performed immunohistochemistry (IHC) on all inflammatory and neurodegenerative brain specimens and quantitative RT-PCR (qPCR) on all NB and tumor samples. In addition, we analyzed patient-derived stem-like GBM cell lines (*n* = 3, kindly provided by S. Brandner, University College London, Division of Neuropathology, London, UK) by IHC. Unfortunately, paraffin-embedded tissue was not available for some of the NB and tumor samples. Therefore, we performed IHC only with 6 NB, 36 gliomas WHO grade II/III, and 62 GBM. In addition, we retrospectively collected clinical data for the WHO grade II/III glioma patients (Table 1) and 43 of the GBM patients (Table 2) for correlation analyses. 

### 2.2. RPS27 Protein Was Expressed by Neurons, Tumor-Associated Macrophages, Astrocytic Tumor Cells, and Stem-Like GBM Cells

**Figure 1:**
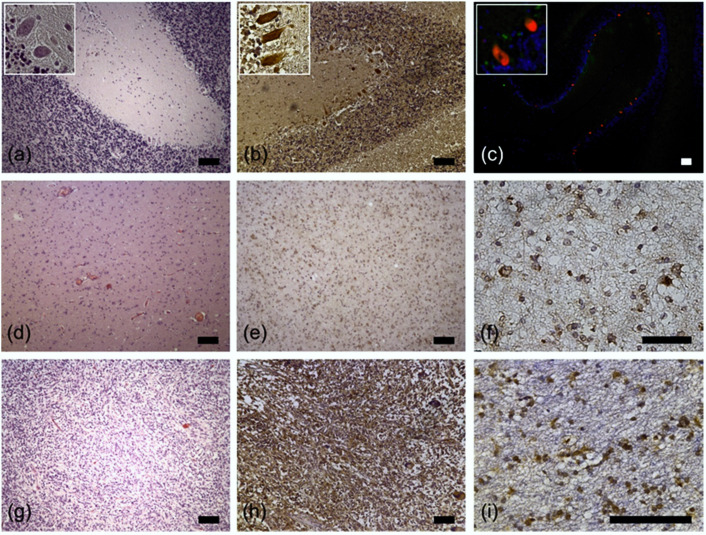
Representative examples of RPS27 immunohistochemistry. (**a**) H&E, (**b**) RPS27-DAB, and (**c**) RPS27 fluorescence staining of normal cerebellar tissue. Purkinje cells were positive for RPS27 staining (red). DAPI = blue. Insets’ objective magnification 40×. (**d**) H&E, (**e**) and (**f**) RPS27-DAB staining of an exemplary WHO grade II glioma. (**g**) H&E, (**h**) and (**i**) RPS27-DAB staining of an exemplary glioblastoma. Scale bars = 100 µm.

First, we aimed to confirm the data published by Fernandez-Pol [27] and performed RPS27-staining of normal cerebellar tissue. Indeed, Purkinje cells strongly stained positive (Figure 1a–c). Based on the finding that U138 GBM cells express RPS27 [28], we then wanted to know whether glioma cells express RPS27 in vivo. We stained glioma WHO grade II/III (Figure 1d–f) and GBM tissue from patients (Figure 1g–i), both revealing clear RPS27 expression by astrocytic tumor cells. Due to this, the point of whether RPS27 could be used as a tumor-cell-specific IHC marker surfaced.

**Figure 2:**
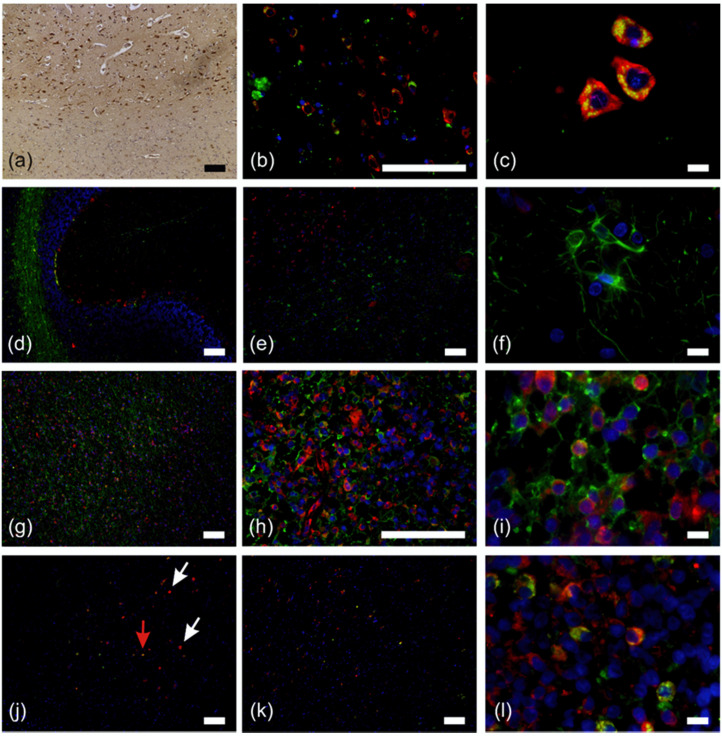
Fluorescence double staining of normal brain and astrocytic tumor specimens. (**a**) RPS27-DAB staining of normal brain tissue. Positive cells were concentrated in the grey matter of the frontal cerebrum. (**b**) and (**c**) neurons (NeuN = green, RPS27 = red) in the frontal cerebrum. (**d**) Overview of the cerebellum double stained for RPS27 (red) and astrocytes (glial fibrillary acidic protein, GFAP = green). (**e**) Neurons and astrocytes in the frontal cerebrum (RPS27 = red, GFAP = green) and (**f**) astrocytes in 100× magnification (RPS27 = red, GFAP = green). (**g**–**i**) Glioblastoma (GBM) cells in the cerebellum. RPS27 = red, GFAP = green. (**j**) Diffuse IDH1 R132H mutant GBM. (RPS27 = red, IDH1 R132H = green). Red arrow = RPS27/IDH R132H double positive tumor cell, white arrows = RPS27-positive but IDH1 R132H-negative cells. (**k**) and (**l**) GBM double staining for RPS27 (red) and macrophages (CD68 = green). DAPI = blue, scale bars in (**c**), (**f**), (**i**), and (**l**) = 10 µm, otherwise scale bars = 100 µm.

Hence, we stained NB tissue for RPS27 (Figure 2a), revealing positive cells concentrated in the grey matter of the frontal cerebrum. These cells were mainly neurons, as we determined by fluorescence double staining for RPS27 in combination with the neuronal marker NeuN (Figure 2b,c). The perikarya of all observed neurons in the cerebellum and cerebrum and some small structures in the white matter, most likely resembling the axons and dendrites, were clearly positive for both markers. However, astrocytes were only detectable by the specific astrocyte marker glial fibrillary acidic protein (GFAP) but were negative for RPS27 (Figure 2d–f). The border between white and grey matter was clearly visible in this staining, due to the RPS27-positive neurons and solely GFAP-stained astrocytes (Figure 2e). In contrast, looking at GBM tissue, double staining of RPS27 with GFAP was obvious, indicating the astrocytic tumor cells (Figure 2g–i). We verified this assumption by double staining an IDH-mutant diffuse GBM for RPS27 and IDH1 R132H (Figure 2j red arrow). However, several cells within these tumors were positive for RPS27, but negative for GFAP as well as IDH1 R132H (Figure 2j white arrows). It is known that up to 50% of the cells within the GBM tumor mass are represented by macrophages [29]. Therefore, whether these cells may indeed be tumor-associated macrophages came into question and double staining for RPS27 was thus performed in conjunction with the macrophage marker CD68 on the same slide, confirming the presence of RPS27-positive macrophages within the tumor (Figure 2k,l).

**Figure 3:**
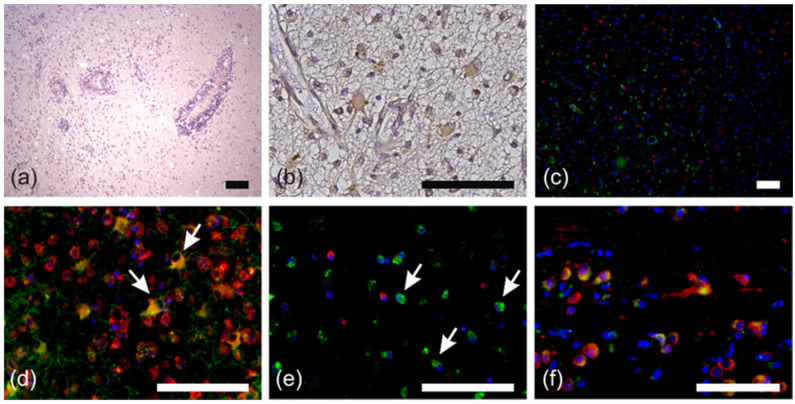
RPS27 staining in multiple sclerosis and encephalitis specimen. (**a**) H&E staining of the cerebellar inflammation in multiple sclerosis tissue. (**b**) RPS27-DAB staining in the cerebrum of multiple sclerosis tissue. (**c**) Immunofluorescence double staining for RPS27 (red) and astrocytes (GFAP = green) in the cerebrum of multiple sclerosis tissue. (**d**) Inflamed astrocytes with RPS27 (red) and GFAP (green) double positivity (arrows). (**e**) Immunofluorescence double staining for RPS27 (red) and macrophages (CD68 = green) in multiple sclerosis tissue. Arrows indicate CD68-positive but RPS27-negative cells. (**f**) Immunofluorescence double staining for RPS27 (red) and macrophages (CD68 = green) in encephalitis. DAPI = blue, scale bars = 100 µm.

This result prompted us to ask whether RPS27 is generally expressed by macrophages during inflammatory processes of the brain. Hence, we stained multiple sclerosis and encephalitis specimen for the markers stated above. In contrast to normal astrocytes, inflammatory astrocytes were weakly positive for RPS27 in both diseases (Figure 3). Interestingly, only a minority of the macrophages were positive for RPS27 (Figure 3). Specimens of the neurodegenerative Alzheimer’s disease confirmed the data on RPS27 expression by Purkinje cells, neurons, and its absence in astrocytes (Figure S1).

**Figure 4:**
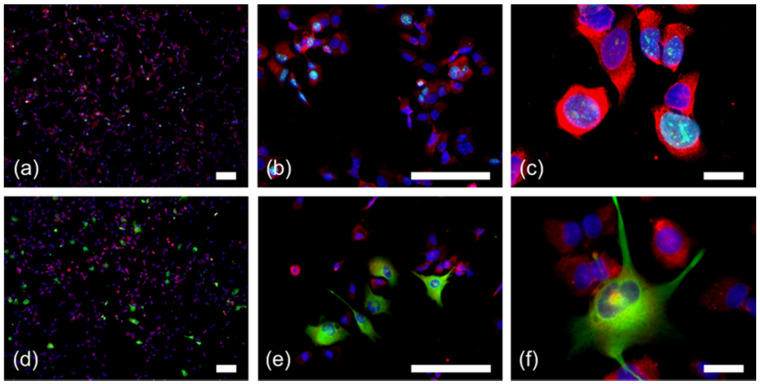
RPS27 staining in conjunction with Ki67 and GFAP positivity in GBM stem-like cells. (**a**–**c**) Immunofluorescence double staining for RPS27 (red) and Ki67 (green). (**d**–**f**) Immunofluorescence double staining for RPS27 (red) and GFAP (green). DAPI = blue, scale bars in (**c**) and (**f**) = 20 µm, otherwise scale bars = 100 µm. For the single color channels see Figure S2.

However, not all RPS27-positive, but GFAP-negative cells within GBM may represent macrophages. Some of these cells may be glioma stem-like cells that lost GFAP expression. Therefore, we analyzed patient-derived stem-like glioma cell lines (Figure 4). As expected, these cells were CD68-negative (Figure S2a), but all of them were positive for RPS27 (Figure 4, Figure S2d,g). The RPS27 expression was independent of the proliferation marker Ki67 (Figure 4a–c, Figure S2c). Some of these cells co-expressed GFAP, while the majority did not (Figure 4d–f, Figure S2f).

Although not suitable as a tumor cell-specific marker protein, our data may be a first hint for a potential role of RPS27 in tumor progression and immunological tumor response. For this reason, we examined whether RPS27 expression correlates with tumor characteristics or patients’ clinical course.

### 2.3. RPS27 was Overexpressed in Gliomas independently of varying Tumor and Patient Characteristics

**Figure 5:**
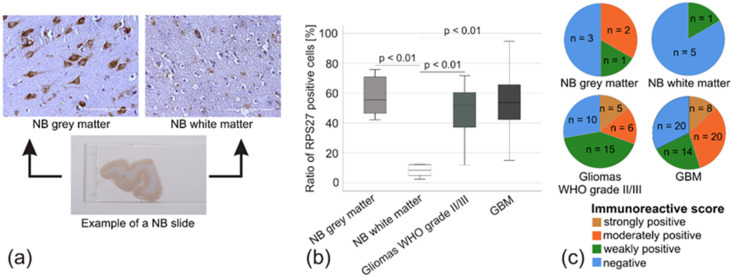
RPS27 protein expression estimated by immunohistochemistry. (**a**) Example of an NB slice with RPS27-DAB staining in the grey and white matter. Scale bars = 100 µm. (**b**) Percentage of RPS27-positive cells compared to all cells. *p*-values based on ANOVA. (**c**) Pie-diagram based on immunohistochemical score.

Macroscopically, it was obvious that RPS27 strongly stained the grey matter containing the neurons’ cell bodies within the NB, whereas the white matter was mainly negative (Figure 5a). Quantification of the stained cells confirmed this assumption. RPS27 expression was significantly stronger in the grey matter, gliomas WHO grade II/III and GBM compared to the NB white matter (all *p*-values < 0.01; Figure 5b). The average optical density, mainly influenced by the background staining, was comparable between the groups, indicating the high specificity of the cells’ staining for RPS27 (Table S1) and allowing us to calculate an immunoreactive score [30]. Five of the 6 NB white matter specimens scored negative, with only one sample weakly positive. NB grey matter, gliomas WHO grade II/III, and GBM were represented by at least 30% of moderately or strongly stained specimens (Figure 5c). Although there was no difference in the tumor samples in staining intensity compared to NB grey matter, these data suggest strong overexpression of RPS27 protein compared to white matter.

**Figure 6:**
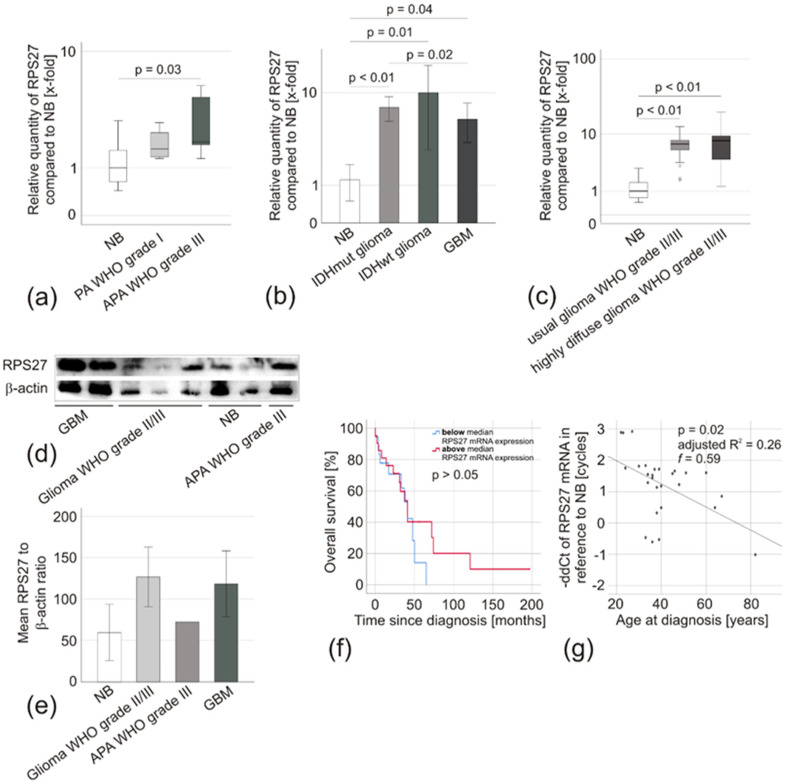
RPS27 expression in glioma specimens. (**a**) Box-plot of RPS27 mRNA expression in pilocytic astrocytoma WHO grade I (PA, *n* = 4) and anaplastic PA WHO grade III (APA, *n* = 5) compared to NB (*n* = 8). The median is displayed by the middle line, the quartiles are represented by the hinges, extreme values up to 1.5 times the height of the box are shown by whiskers. Differences of ddCt-values were compared by ANOVA, Leven’s test used to assess the equality of variances, and Scheffe procedure or Dunnet T3 chosen as posthoc tests. (**b**) Relative RPS27 mRNA expression compared to NB (*n* = 8). (IDHmut glioma *n* = 28, IDHwt glioma *n* = 13, GBM *n* = 76). (**c**) Box-plot of RPS27 mRNA expression in usually growing (infiltration into 1-3 lobes, *n* = 17) and highly diffuse (infiltration into > 3 lobes) gliomas WHO grade II/III (*n* = 41) compared to NB (*n* = 8). (**d**) Western blot of randomly selected samples of each tumor entity and (**e**) quantification by densitometry in relation to the housekeeping gene β-actin. The unchanged original blots are shown in Figure S3. (**f**) Kaplan–Meier analysis comparing the overall survival of WHO grade II/III glioma patients with RPS27 mRNA expression below (blue, *n* = 21) and above (red, *n* = 20) the median expression of 7.9-fold by Breslow test. (**g**) Scatter diagram of RPS27 mRNA expression compared with the age of WHO grade II/III glioma patients. Linear regression, p-value based on ANOVA, effect size based on Cohen’s f. IDHmut glioma and IDHwt glioma were combined for the analyses presented in (**c**), (**d**), and (**e**).

Next, we inquired whether these results are also reflected by RPS27 mRNA expression and might correlate with the clinical parameters of the patients. Analyzing RPS27 expression in the Gutman Brain dataset [31] did not reveal overexpression in PA. We observed a significantly enhanced copy number of the RPS27 gene in oligodendroglial tumors WHO grade III by the Oncomine analysis tool based on the ‘The Cancer Genome Atlas’ (TCGA) brain 2 dataset (*p* < 0.01). Unfortunately, our ethics vote did not allow the evaluation of tumor samples from these entities. However, we analyzed 4 adult (age > 18 years) PA WHO grade I and 5 APA WHO grade III for RPS27 mRNA expression. Compared to NB, there was no significant overexpression in PA WHO grade I, while a mean 1.9-fold overexpression was observed in APA WHO grade III (ddCT = 1.4, *p* = 0.03) (Figure 6a). Similarly, RPS27 mRNA was overexpressed in IDHmut glioma (mean expression 6.2-fold, *p* < 0.01), IDHwt glioma (mean expression 8.8-fold, *p* = 0.01), and in GBM (mean expression 4.6-fold; *p* = 0.04). The latter was confirmed by analyzing the TCGA brain dataset in Oncomine, which compared 10 NB with 542 GBM and revealed a 1.274-fold change in RPS27 mRNA expression (*p* < 0.01). RPS27 mRNA expression in IDHmut glioma was slightly, but significantly higher than in GBM (*p* = 0.02) (all p-values based on ddCT) (Figure 6b). Within the gliomas of WHO grade II/III (IDHmut glioma and IDHwt glioma combined) subgroup, there was no difference in RPS27 mRNA expression between usually growing (infiltration into 1–3 lobes, mean expression 7.5-fold) and highly-diffuse tumors (infiltration into more than three lobes, mean expression 6.7-fold) (Figure 6c). A very similar expression, as seen for RPS27 mRNA, was confirmed on protein levels by Western blot (Figure 6d,e). IDHmut glioma patients with an RPS27 expression above or below the median expression of 7.9-fold had a comparable overall survival (OS) (*p* > 0.05) (Figure 6f). Interestingly, younger age was associated with a significantly higher RPS27 expression determined by both regression (Figure 6g) and correlation analyses (Table 3).

**Figure 7:**
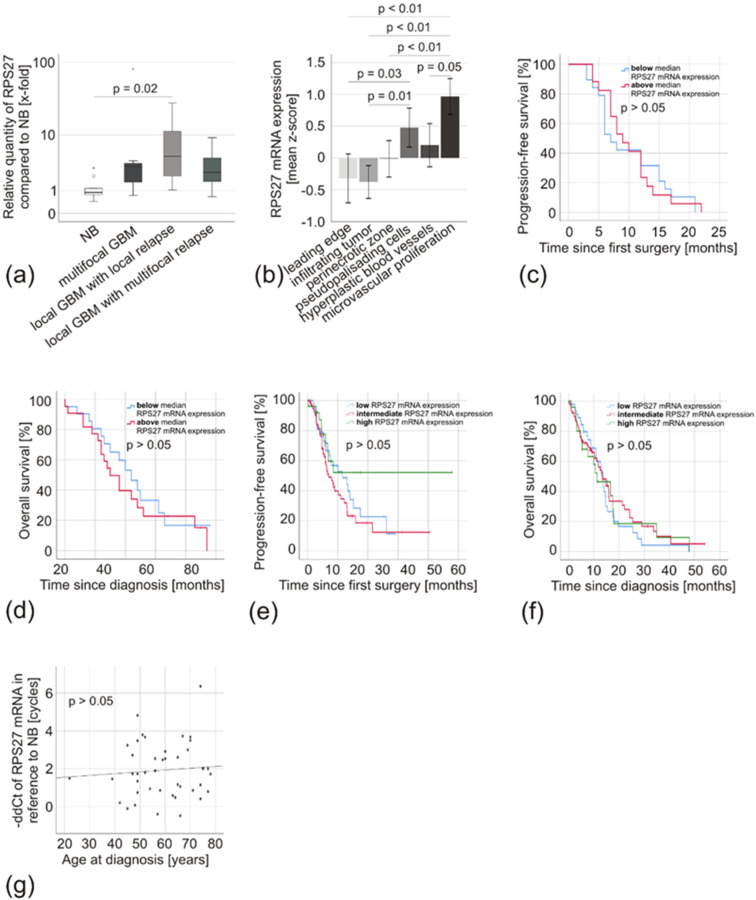
RPS27 mRNA expression in GBM specimens. (**a**) Box-plot of RPS27 mRNA expression of primary GBM with different growth patterns (multifocal GBM *n* = 7, local GBM with local relapse *n* = 24, local GBM with multifocal relapse *n* = 12) compared to NB (*n* = 8). The median is displayed by the middle line, the quartiles are represented by the hinges, extreme values up to 1.5 times the height of the box are shown by whiskers and outliners represented by points. (**b**) IVY-GAP database [32] analysis of RPS27 mRNA expression in different areas of GBM: leading edge (*n* = 19), infiltrating tumor (*n* = 24), perinecrotic zone (*n* = 26), pseudopalisading cells around necrosis (*n* = 40), hyperplastic blood vessels in cellular tumor (*n* = 22), and microvascular proliferation (*n* = 28). (**c**) Kaplan–Meier analysis comparing the progression-free and (**d**) the overall survival of GBM patients with RPS27 mRNA expression below (blue, *n* = 22 and *n* = 21, respectively) and above (red, *n* = 17 and *n* = 22, respectively) the median expression of 2.7 fold by Breslow test. (**e**) Kaplan–Meier analysis comparing the progression-free and (**f**) the overall survival of GBM patients with low (blue, *n* = 47), intermediate (red, *n* = 78) and high (green, *n* = 26) RPS27 mRNA expression. TCGA data were exported from the CBioportal analysis tool [33]. (**g**) Scatter diagram of RPS27 mRNA expression compared with the age of GBM patients. Linear regression, *p*-value based on ANOVA.

Looking at GBM, very similar results could be obtained. Independent of their growth pattern (primary multifocal tumors, mean expression 12.7-fold due to a high outliner, median expression 3.3-fold; primary local tumors with local relapse, mean expression 5.6-fold; and primary local tumors with multifocal relapse, mean expression 2.3-fold) there was no difference in RPS27 mRNA expression (*p* > 0.05) (Figure 7a). We detected no significant difference between primary tumors and their respective relapses (*p* > 0.05; primary local GBM with local relapse, mean expression 5.6-fold vs. their local relapse, mean expression 4.7-fold; primary local GBM with multifocal relapse, mean expression 2.3-fold vs. their multifocal relapse, mean expression 14.3-fold). The IVY Glioblastoma Atlas Project (IVY-GAP) database allows examining regional differences in mRNA expression within the tumor [32]. It revealed enhanced RPS27 expression in microvascular proliferation and pseudopalisading cells around necrosis, but not at the leading edge and by infiltrating tumor cells (Figure 7b). We did not see any difference in the progression-free survival (PFS) or the OS of patients with RPS27 mRNA expression above or below the median 2.7-fold expression (*p* > 0.05) (Figure 7c,d), which was confirmed by analyzing TCGA data with the CBioportal analysis tool (Figure 7e,f) [33]. In addition, there was no correlation with other clinical characteristics including the patients’ age (Figure 7g), except for a weak correlation with Ki67 staining (r = 0.33, *p* = 0.04, Table 4).

## 3. Discussion

RPS27 is a ribosomal protein. Due to its ubiquitous and stable expression level throughout different tissues and cell types, it has been suggested to be a housekeeping gene, which might be useful as a reference in gene expression studies [34]. However, it is not only involved in protein synthesis, but as a multifunctional protein, it also plays a role in regeneration and oncogenesis. Indeed, it is overexpressed in a multitude of human cancers [20,21,22,23,24,25]. Fernandez-Pol [27] described RPS27-expression in Purkinje cells of the cerebellum [27], a result we could confirm, and it is known to be expressed in a GBM cell line [28]. Otherwise, data on RPS27 in the CNS are surprisingly limited. There is, to the best of our knowledge, no report on the expression of RPS27 in gliomas of different WHO grades. Thus, we aimed to identify RPS27 expressing cells in the CNS with special emphasis on astrocytic tumors. We observed RPS27 expression in all neurons examined. Interestingly, RPS27 was not detectable in normal astrocytes, but clearly present in astrocytic tumor cells. Therefore, we determined whether it might be suitable as a tumor-specific staining marker and compared its expression in gliomas with those in neurodegenerative and inflamed tissue. Unfortunately, RPS27 also weakly stained astrocytes in inflammatory tissue and thus cannot be used as a tumor-specific staining marker. To our surprise, the CD68/RPS27 double staining indicated another difference between tumor and non-tumor tissue. Almost all macrophages in tumor tissue were distinctly positive for RPS27, whereas only a minority of macrophages in inflammatory tissue was. RPS27 positivity has been observed for macrophages associated with melanomas [35]. Since those macrophages in tumor areas with high RPS27 expression were intensely positive and those associated with less intensely stained tumor areas had less RPS27 content, it was discussed whether its abundant presence in tumor-associated macrophages may be related to direct phagocytosis of RPS27-positive cell debris [35]. On the other hand, we now know that macrophages exist in different phenotypes. Their function differs between inflammation and tumor [36]. Our data suggest, that RPS27 might be either involved in or influenced by these shifts of macrophage configuration. It seems promising, to further evaluate this association.

Although tumor-associated macrophages represent up to 50% of the GBM cell mass [29], this staining did not exclude the possibility of another RPS27-positive, but GFAP- and CD68-negative cell type present within the tumor. Indeed, glioma stem-like cells expressed RPS27, which was independent of GFAP or Ki67 positivity. Glioma stem-like cells to a large extent contribute to therapy resistance and recurrence of gliomas [37]. Whether RPS27 might be involved in these processes remains a so far unmet question.

Thus, our immunofluorescence staining supported the hypothesis of RPS27 being dysregulated in gliomas. Therefore, we quantified its expression and confirmed the suspected overexpression of RPS27 on protein level by DAB staining and Western blotting in gliomas of WHO grade II/III and in GBM. On the mRNA level, we confirmed the overexpression of RPS27 in IDHmut glioma, IDHwt glioma, and GBM, which was further validated by analyzing TCGA data. The IVY-GAP database [32] revealed that its expression was regionally enhanced in microvascular proliferation and in pseudopalisading cells around necrosis. These cells represent a wave of hypoxic tumor cells, which are less proliferative and actively migrating away from central hypoxia [38]. In addition, they are responsible for inducing nearby angiogenesis by secretion of angiogenic factors such as VEGF [38]. The expression of RPS27 in both pseudopalisading cells and microvascular proliferation might perhaps indicate an involvement of RPS27 in these processes. However, despite a striking fluctuation range, this overexpression was independent of clinical or molecular characteristics of the patients, such as the tumor growth pattern, and it was not associated with a difference in PFS or OS. Since mRNA expression does not necessarily reflect protein expression due to post-translational modification, we cannot exclude that there might be a correlation of RPS27 protein expression with the patients’ clinical course. Nevertheless, TCGA data largely confirmed our findings, except for a recent report analyzing the TCGA dataset showing that patients diagnosed with low-grade gliomas and high RPS27 expression had a better prognosis than those patients with low expression [39]. Having a second look at our Kaplan–Meier analysis, we see a similar trend for the long-term survivors in our WHO grade II/III cohort.

MGMT promoter methylation usually is seen in about 35% of the general GBM population [40]. Although in our cohort, the methylation seems to be quite high (69%), it is in the range of other reports [18,41]. Nevertheless, the high proportion of methylated patients may also be due to a selection bias. When assembling our cohort, we chose specifically patients with matched samples from their primary tumor and relapse. Therefore, we may have chosen patients with longer survival. Long-term surviving patients have an increased probability of bearing a methylated MGMT promoter [8].

Interestingly, expression in GBM was lower than in IDHmut glioma and IDHwt glioma. One possible explanation might be that GBM patients are typically older than their IDHmut glioma counterparts, which coincides with our finding of younger patients having significantly higher RPS27 levels in IDHmut gliomas. However, the observed correlation is probably just a statistical coincidence, because we could not make a similar observation in GBM, and CBioportal analyses showed only a slight tendency for RPS27 expression in younger patients. On the other hand, the observed effect on IHDmut gliomas was mostly based on high expression in patients below the age of 50, while our GBM collective mainly consisted of patients older than 50 years. Although age-dependent regulation of RPS27 has not been described so far, strong RPS27 expression could promote malignant transformation, possibly leading to earlier glioma development in young patients. For melanoma, it has been discussed that cells with high RPS27 expression were characterized by higher proliferation and invasion capabilities, whereas RPS27 low cells displayed a reduced adherence, higher anchorage-independent survival, and more aggressive and drug-resistant behavior [39]. RPS27 is integrated into the NF-κB, JNK/Jun, MAPK/ERK, and p53 signaling pathways, which are all important for tumorigenesis or therapeutic resistance [27,42,43,44,45,46,47]. Nevertheless, the exact regulatory and signaling network controlling and involving RPS27 and its role in glioma stem-like cells, tumor-associated macrophages, and glioma tumor cells requires further clarification.

In summary, we found RPS27 overexpressed in astrocytic tumors, independently of their WHO grade, IDH-mutation status, or tumor/patient characteristics. Its association with tumor cells may be a first hint for a potential role during gliomagenesis, a conclusion supported by the multitude of similar reports on other tumor entities [20,21,22,23,24,25,47,48]. RPS27 is released from the tumor by secretion or gradient diffusion and can, therefore, be measured in the patients’ serum [24,26,49,50,51]. It could be valuable as a liquid biopsy marker for tumor- and recurrence detection, a promising aspect we are currently examining for GBM patients.

## 4. Materials and Methods

### 4.1. Tissue Samples and Clinical Data

We procured tissue samples from patients treated in the Department of Neurosurgery, University Hospital Würzburg, Germany. The patients included in this study stated their written informed consent in accordance with the International Conference on Harmonization, the declaration of Helsinki, as approved by the Institutional Review Board of the University of Würzburg (#103/14). In addition, we obtained autopsy/biopsy samples of non-pathological brain tissue (normal brain, NB), inflammatory brain tissue (multiple sclerosis, encephalitis), and neurodegenerative brain tissue (Alzheimer’s disease) from the Brain Bank of the Department of Neuropathology, Institute of Pathology, University of Würzburg, Germany (approval #78/99). After acquisition, we froze half of the tissue specimens at −80 °C for RNA-analysis and formalin-fixed, as well as paraffin-embedded, the other half for immunohistochemistry. In case the available tissue was too little to be used for all experiments, we put emphasis on collecting fresh-frozen samples. We classified the tumors by routine histology based on WHO criteria [1], and only included tumor samples if the estimated tumor cell content was at least 80%. We reconstructed the clinical course of the patients retrospectively by collecting information such as tumor localization, growth pattern (local/multifocal), treatment, recurrence, and outcome. Finally, we determined known prognostic factors, like extent of surgical resection by evaluation of post-operative Magnetic Resonance Imaging (MRI) images, tumor-volume by semiautomatic tumor-volumetry on MRI images, IDH mutation status by immunohistochemistry and pyrosequencing, or MGMT promoter methylation by high-resolution melting real-time polymerase chain reaction, as described previously [52].

### 4.2. Glioma Stem-Like Cell Culture

We cultured three patient-derived GBM stem-like cell lines, kindly provided by S. Brandner (University College London, Division of Neuropathology, London, UK) in 6-well culture plates at 37 °C, 5% CO_2_, and 100% humidity in NeuroCult NS-A basal medium supplemented with NeuroCult NSA proliferation supplement (1:10), 0.2% Heparin (1:1000), Hu Recom EGF (1:5000), Hu Recom bFGF (1:10,000; all from STEMCELL Technologies, Vancouver, Canada), laminin from Engelbreth-Holm Swarm murine sarcoma basement membrane (1:1000; Sigma Aldrich, St. Louis, MO, USA), and penicillin-streptomycin (1:100, 10,000 u/mL penicillin, 10,000 µg/mL streptomycin; Thermo Fisher Scientific, Waltham, MA, USA). We split the cells when they reached 80–90% confluency by collecting the media supernatant, washing the cells once with phosphate buffered saline (PBS; Biochrom, Berlin, Germany), collecting the PBS as well and then detaching the cells with Accutase (STEMMCELL Technologies, Vancouver, Canada). The detached cells and collected fluids were centrifuged for 10 min at 200× *g*. We discarded the supernatant, resuspended the cells in 1 mL medium and counted them using the Countess II Automated Cell Counter (Thermo Fisher Scientific, Waltham, MA, USA) before seeding 20,000 of them for staining experiments onto laminin-coated coverslips.

### 4.3. RNA Extraction and Quantitative RT-PCR

We extracted the mRNA from frozen tissue samples by using the TRIzol^®^ Reagent (Thermo Fisher Scientific, Waltham, MA, USA) and converted the mRNA to DNA as previously described [52]. We evaluated the relative RPS27 mRNA expression in a duplex setting utilizing the StepOnePlus Real-time PCR System, TaqMan Universal PCR Master Mix, GAPDH_VIC_PL (Hs99999905_m1) as internal control and RPS27_FAM (Hs01378332_g1) as probe (all from Applied Biosystems, Foster, CA, USA). We ran all samples in triplets with 20 ng DNA each. The PCR’s cycling started at 50 °C for 2 min, followed by 10 min at 95 °C, and 50 cycles of 15 s at 95 °C and 1 min at 60 °C. In case a standard deviation exceeded 0.5 Ct in triplets, we repeated the PCR. Biopsy- and autopsy-derived NB tissue was treated as a combined group as they displayed similar mRNA-expression (*p* > 0.05).

### 4.4. Western Blot Analysis

Cells were washed twice with PBS and lysed with RIPA buffer (50 mM Tris pH 8.0, 150 mM NaCl, 0.1% SDS, 0.5% sodium deoxycholate, 1% NP40) containing protease inhibitor cocktail (Roche, Mannheim, Germany) and Phenylmethylsulfonylfluoride (PMSF). Cells were afterwards sonicated (Bandelin SONOPULS) and mixed with Laemmli buffer containing 5% β-mercaptoethanol. After denaturation at 95 °C, they were run through a 15% SDS PAGE mini gel and blotted overnight using a Mini Trans-Blot Electrophoretic Transfer Cell (BioRad, Hercules, CA, USA). Subsequently, the membrane was blocked in 5% non-fat dry milk (Carl Roth, Karlsruhe, Germany) and probed with the primary MPS1 polyclonal antibody (Table S2) followed by a secondary antibody (Table S2). β-actin served as endogenous control (Table S2). Detection was carried out using an electrochemiluminescence solution [53] and viewed with ImagenFlourChem FC2 (Cell Biosciences, Heidelberg, Australia) with the AlphaView Software (Version 1.3.0.7, ProteinSimple, San Jose, CA, USA). Densitometric analysis was carried out using Image J (NIH, Bethesda, MD, USA).

### 4.5. Immunohistochemistry (IHC)

To prepare tissue sections, we cut 3 µm thick slices from formalin-fixed, paraffin-embedded tissue blocks, dewaxed twice with xylene and then rehydrated them in a graded series of ethanol (100%, 96%, 70%; diluted in distilled water), as well as in distilled water. Samples were then processed either by hematoxylin-eosin staining (H&E), 3,3-diaminobenzidine (DAB) IHC, or double-fluorescence staining. All staining procedures and evaluations were supervised by an experienced neuropathologist. Normal cerebellar tissue with specific staining of Purkinje cells as described by Fernandez-Pol [27] served as positive controls. Stains under omission of primary antibody and RPS27-negative cells in the cerebellar tissue (e.g., granule cells) were used as internal negative controls.

For H&E-staining, we incubated the specimens for 10 min with hemalum solution acid acc. to Mayer (Roth, Karlsruhe, Germany), rinsed them with tap water for 10 min, counterstained for 5 min with 1% eosin in aqueous solution (Roth, Karlsruhe, Germany), and passed them through a graded series of ethanol (70%, 96%, 100%) and 100% xylol before embedding them with Eukitt (ORSAtec, Bobingen, Germany).

For DAB-IHC, we performed heat-induced epitope retrieval at 120 °C in citrate buffer (pH = 6.0, 20 mM) for 10 min. To eliminate human peroxidase activity, we then treated the slices with 0.7% hydrogen peroxide, blocked them with 10% normal goat serum (Invitrogen, Carlsbad, CA, USA) and stained them using the Envision System HRP DAB (DAKO, Jena, Germany) and the antibodies listed in Table S2. We counterstained the cell nucleus using hemalum solution acid acc. to Mayer (Roth, Karlsruhe, Germany).

We scored tumors that strongly stained for IDH1R132H in the cytoplasm as positive and evaluated the Ki67 staining based on the percentage of cells with specific positivity. To analyze PRS27 protein expression, we photographed five representative areas of each sample’s RPS27 immunostaining with a LEICA DMI 3000 B microscope, LEICA DFC450 camera, and LAS V4.5 software (all Leica, Wetzlar, Germany) and standardized settings (camera objective: 40×; exposure: 25 ms; gain: 1.0×; gamma: 1; otherwise standard settings). We semi-automatically evaluated the pictures in Fiji [54,55] by processing the macro shown in Table S3.

Briefly explained, we used color deconvolution [56] to segregate hemalum and DAB staining. Subsequently, the intensity of the 8-bit DAB pictures was measured, and the mean intensity was converted into optical density (OD) by the formula OD = log(255/mean intensity). Additionally, the macro counted stained cells in the DAB and hemalum pictures based on a manual threshold set by including the cells and excluding the background staining and the steps listed in above macro. We assessed the staining intensity by means of the immunoreactive score, mainly used for breast cancer [30]. Therefore, we categorized macro obtained OD and cell count into four groups based on the median and quartile values and then calculated the immunoreactive score with these values as described in [30].

For double fluorescence staining, we washed the patient-derived glioma stem-like cells, grown over night on laminin-coated coverslips, with Tween20 wash buffer (1:1000 in PBS, Carl Roth, Karlsruhe, Germany). Subsequently, we fixed the cells with paraformaldehyde (PFA, 1:25 in PBS, pH 7.4; Merck, Darmstadt, Germany) for 10 min at room temperature and washed them three times with ice-cold PBS. We permeabilized the cells’ membranes for 10 min with TritonX-100 (1:400 in PBS, Sigma-Aldrich, St. Louis, MO, USA) at room temperature and washed them 3 times for 5 min with Tween20 wash buffer. Finally, we blocked the samples in Tween20 wash buffer containing bovine serum albumin (1:100, Sigma-Aldrich, St. Louis, MO, USA).

We cautiously heated the tissue slices in 10 mM citrate buffer pH 6 containing 0.05% Tween20 for 20 min and left them in the slowly cooling buffer for another 20 min before placing them into distilled water followed by Trishydroxymethylaminoethan buffer (0.05 M Trishydroxymethylaminoethan, 0.15 M NaCl and 0.1% Tween20 in distilled water, pH 7.6) for 2 min each. Finally, we blocked the specimens in 10% goat serum for 20 min at room temperature. We incubated both tissue sections and stem-like cells overnight with the respective primary antibodies (Table S2) at 4 °C. The next day, we washed the slices 3× in TBST (tissue) and Tween20 wash buffer (cells) for 5 min each and incubated them with the matching secondary antibodies at room temperature for one hour. Again, we washed the samples three times in TBST (tissue) and Tween20 wash buffer (cells) for 5 min each and counterstained them with Fluoroshield, containing DAPI (Abcam, Cambridge, UK). We photographed the slices with the LEICA microscope, using three different filters (Filtercubes A, L5 ET, and Y3 ET, all from Leica, Wetzlar, Germany) and processed them with Fiji [54]. We converted the pictures to 8-bit greyscale images and adjusted their brightness and the contrast individually. This procedure was performed in random order and the investigators were blinded to diagnosis and staining-antibodies of the slides to exclude a potential bias. Finally, the images were reconverted to RGB and merged to an overlay utilizing the image calculator operation: ‘add’.

### 4.6. Bioinformatics and Statistics

Apart from our own patient collective, we analyzed RPS27 mRNA expression, copy number variations, and their associations to patients’ clinical data on TCGA datasets, the Gutman Brain dataset, and the IVY-GAP database [32] (https://glioblastoma.alleninstitute.org) in March 2020. We performed the statistical analysis with IBM SPSS Statistics 23 (IBM Corporation, Armonk, NY, USA) and used the web-based analysis tools CBioportal (https://www.cbioportal.org/) and Oncomine (https://www.oncomine.org) to obtain preprocessed versions of the publicly available datasets. Where required, we normalized dCt-values to the expression of the housekeeping gene GAPDH by the ddCt-method [57]. However, though *p*-values were calculated based on the ddCt-values, boxplots visualize the relative quantity acquired from the ddCt-values. We compared the expression with ANOVA (Levene’s test, Post-hoc: Scheffe-test or Dunnet T3). To determine associations between RPS27 and the chosen tumor/patient characteristics, we performed a non-parametric correlation test (Spearman’s Rho). We compared the outcome by Kaplan–Meier (Breslow test). Due to the low number of samples, we refrained from survival analysis of IDHwt glioma.

## 5. Conclusions

Although RPS27 is overexpressed in various proliferating tissues, as well as a multitude of human malignancies, we are the first to systematically analyze its expression in human glioma tissue. We found its overexpression in glioma WHO grade II/III and in GBM compared to NB on mRNA and protein level. Although RPS27 expression levels did not affect the patients’ survival, its association with tumor cells and tumor-associated macrophages provides a rationale for a future investigation of its potential function during gliomagenesis and tumor immune response.

## Figures and Tables

**Table 1:** Clinical parameters of IDHmut (*n* = 28) and IDHwt glioma (*n* = 13) patients.

Patients’ Characteristics	IDHmut Glioma	IDHwt Glioma
Sex	female: 10/35.7% male: 18/64.3%	female: 4/30.8% male: 9/69.2%
Age (median, quartiles)	38.0 years (33.0–46.0 years)	45.0 years (28.5–57.5 years)
OS (median, quartiles)	31.0 months (8.0–41.0 months)	32.0 months (12.0–49.0 months)
Growth pattern	usual: 11/39.3% highly diffuse: 17/60.7%	usual: 6/46.2% highly diffuse: 7/53.5%

Given are the absolute numbers of the specified glioma patients in each group and the percentage of the analyzed population. Usual growth is defined as the infiltration of 1–3 lobes and highly diffuse growth as the infiltration of 3 or more lobes. OS = overall survival; IDH = isocitrate dehydrogenase; IDHmut glioma = IDH-mutated tumors with the histological appearance of WHO grade II and III gliomas; IDHwt gliomas = IDH wildtype tumors with the histological appearance of WHO grade II and III gliomas.

**Table 2:** Clinical parameters of glioblastoma multiforme (GBM) patients (*n* = 43).

Patients’ Characteristics	
Sex	
female	18/41.9%
male	25/58.1%
Age (median, quartiles)	59.0 years (49.0–70.0 years)
ECOG	
0	22/51.2%
1	17/39.5%
>1	4/9.3%
**Tumor Characteristics**	
Volume (median, quartiles)	30.1 cm^3^ (16.2–54.3 cm^3^)
Localization	
left hemisphere	24/55.8%
right hemisphere	16/37.2%
both hemispheres	3/7.0%
Localization (lobe)	
frontal	13/30.2%
occipital	5/11.6%
temporal	7/16.3%
parietal	5/11.6%
multiple	13/30.2%
MGMT promoter methylation ^1^	
unmethylated	10/31.3%
methylated	22/68.7%
Ki67 staining (median, quartiles)	25% (20–30)
**Therapy**	
Time from diagnosis to therapy	
0–7 days	27/62.9%
8–14 days	9/20.9%
>14 days	7/16.2%
Surgical intervention	
biopsy	6/14.0%
complete resection	9/20.9%
incomplete resection	28/65.1%
Radiation therapy	
yes	40/93.0%
no	3/7.0%
TMZ chemotherapy	
yes	35/81.4%
no	8/18.6%
**Relapse and Outcome**	
PFS ^2^	
0–6 months	2/5.6%
>6 months	34/94.4%
Relapse	
primarily multifocal	7/16.3%
local	24/55.8%
multifocal	12/27.9%
OS	
0–6 months	4/9.3%
>6 months	39/90.7%

Given are the absolute numbers of the GBM patients in each group and the percentage of the analyzed population. ^1^ Due to a lack of sufficient tissue samples, the MGMT promoter methylation status could not be re-evaluated for some patients. ^2^ Some patients did not match criteria for tumor progression and, therefore, were excluded from the PFS analysis. ECOG = Eastern Cooperative Oncology Group score; MGMT = O6-methylguanine-DNA methyltransferase; TMZ = Temozolomide; PFS = progression-free survival; OS = overall survival.

**Table 3:** Correlation of IDHmut glioma patients’ characteristics with RPS27 mRNA expression.

Patient Characteristics	Correlation Coefficient *r*	*p*-Value	Statistical Test
Sex (female/male)	n.d.	0.676	ANOVA
**Age**	**0.519**	**<0.01**	Non-parametric correlation
OS	−0.214	0.294	Non-parametric correlation
Growth pattern (usual/highly diffuse)	n.d.	0.520	ANOVA

Correlation of RPS27 mRNA expression with patients’ characteristics was examined by non-parametric tests (Spearman’s Rho). Expression differences were examined by ANOVA (ddCT-values, posthoc test: Scheffe procedure or Dunnet T3). Significant results are shown in bold. IDHmut glioma = isocitrate dehydrogenase mutant astrocytic tumor with the histological appearance of WHO grade II and III gliomas; OS = overall survival; n.d. = not determined.

**Table 4:** Correlation of GBM patient and tumor characteristics with RPS27 mRNA expression.

Patient and Tumor Characteristics	Correlation Coefficient *r*	*p*-Value	Statistical Test
Sex (female/male)	n.d.	0.47	ANOVA
Age	-0.07	0.66	Non-parametric correlation
ECOG (median)	n.d.	0.42	ANOVA
Tumor volume	0.01	0.93	Non-parametric correlation
Tumor localization (left/right/both hemispheres)	n.d.	0.90	ANOVA
Tumor localization (lobe: frontal/occipital/temporal/parietal/multifocal)	n.d.	0.27	ANOVA
MGMT promoter methylation (unmethylated/methylated)	n.d.	0.43	ANOVA
**Ki67 staining**	**0.33**	**0.04**	Non-parametric correlation
Surgical intervention	n.d.	0.79	ANOVA
(biopsy/incomplete resection/complete resection) Tumor growth pattern (multifocal/local relapse [primary tumor/relapse]/multifocal relapse [primary tumor/relapse])	n.d.	0.84	ANOVA
PFS	0.04	0.83	Non-parametric correlation
OS	-0.15	0.33	Non-parametric correlation

Correlation of RPS27 mRNA expression with selected patient and tumor characteristics were examined by non-parametric tests (Spearman’s Rho). Expression differences were examined by ANOVA (ddCT-values, posthoc test: Scheffe procedure or Dunnet T3). Significant results are shown in bold. ECOG = Eastern Cooperative Oncology Group scale; n.d. = not determined; MGMT = O6-methylguanine DNA-methyltransferase; PFS = progression-free survival; OS = overall survival.

## Supplementary Materials

The following are available online at https://www.mdpi.com/2072-6694/12/5/1085/s1, Figure S1: RPS27 staining in Alzheimer’s disease, Figure S2: RPS27, CD68, Ki67 and GFAP staining in GBM stem-like cells, Figure S3: Unaltered Western blot of RPS27 and β-actin protein expression in randomly selected glioma samples, Table S1: Average optical density (OD) of DAB-stained NB and tumor specimens analyzed in Figure 5, Table S2: Antibodies used for immunohistochemistry and double-fluorescence staining, Table S3: Macro used for semi-automatic picture evaluation.
