# Supplementary material for: Ribosomal Protein S27/Metallopanstimulin-1 (RPS27) in Glioma—A New Disease Biomarker?
Source: Cancers (Basel). 2020 Apr 27;12(5):1085. doi: 10.3390/cancers12051085 (PMC7281545; doi:10.3390/cancers12051085)
Supplement: Supplementary file 1 [file cancers-12-01085-s001.pdf]

Supplementary Materials: Ribosomal Protein S27/Metallopanstimulin-1 (RPS27) in Glioma–A New Disease Biomarker?

Jonas Feldheim, Almuth F. Kessler, Dominik Schmitt, Ellaine Salvador, Camelia M. Monoranu, Julia J. Feldheim, Ralf-Ingo Ernestus, Mario Löhr and Carsten Hagemann

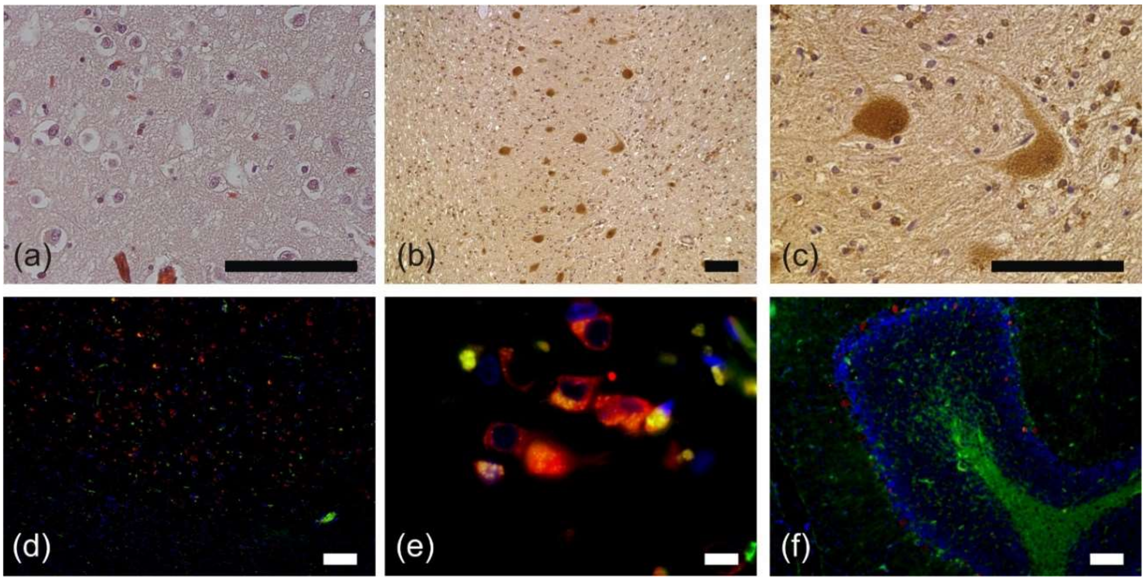

Figure S1. RPS27 staining in Alzheimer's disease. (**a**) H&E staining of the cerebrum of an Alzheimer's patient. (**b**) and (**c**) RPS27-DAB staining in the cerebellum. Neurons were positive. (**d**) and (**e**) Immunofluorescence double staining for RPS27 (red) and neurons (NeuN = green) in the cerebrum of an Alzheimer's patient. (**f**) Immunofluorescence double staining for RPS27 (red) and astrocytes (GFAP = green) in the cerebellum of an Alzheimer's patient. DAPI = blue, scale bars = 100 μm.

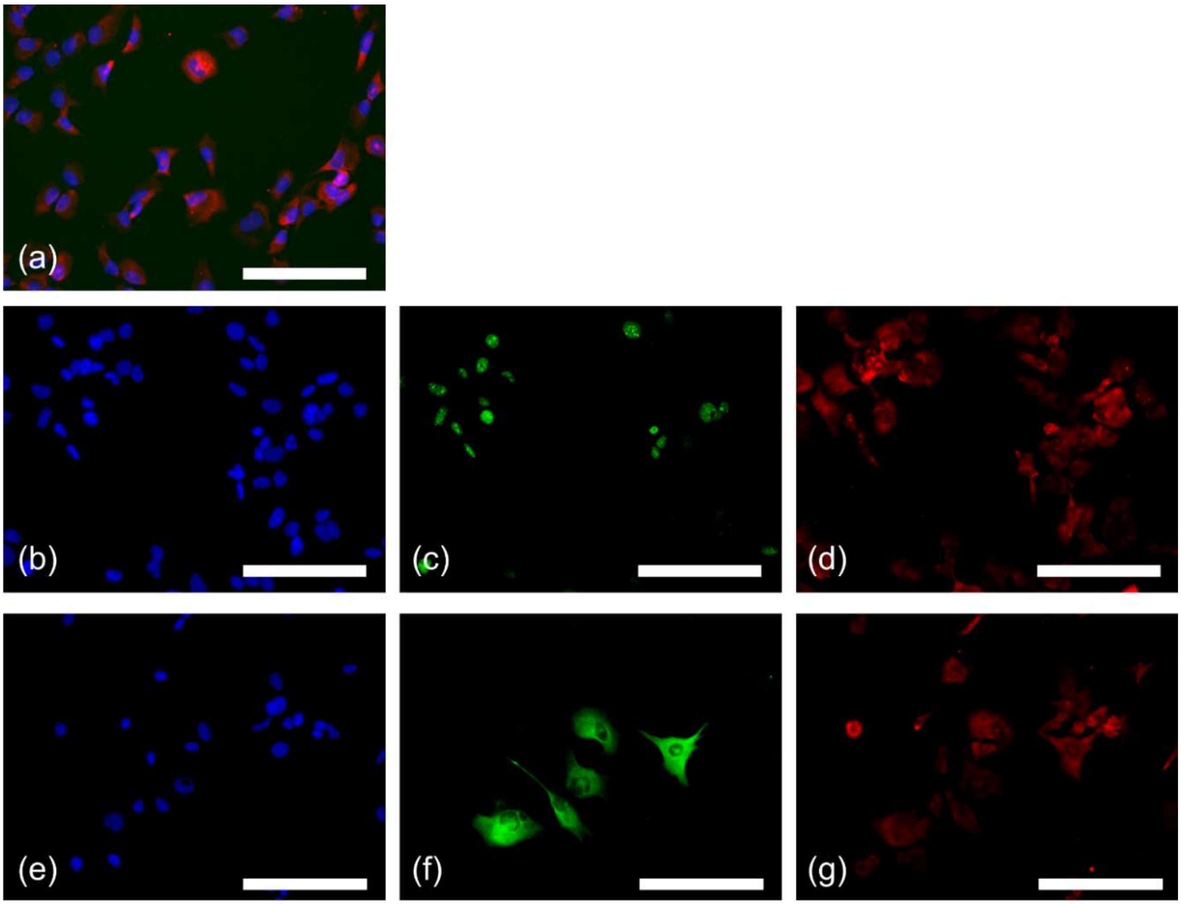

Figure S2. RPS27, CD68, Ki67 and GFAP staining in GBM stem-like cells. (**a**) Immunofluorescence double staining for RPS27 (red) and CD68 (green). (**b**) DAPI (blue), (**c**) Ki67 (green) and (**d**) RPS27 (red) immunofluorescence staining. (**e**) DAPI (blue), (**f**) GFAP (green) and (**g**) RPS27 (red) immunofluorescence staining. Scale bars = 100 μm.

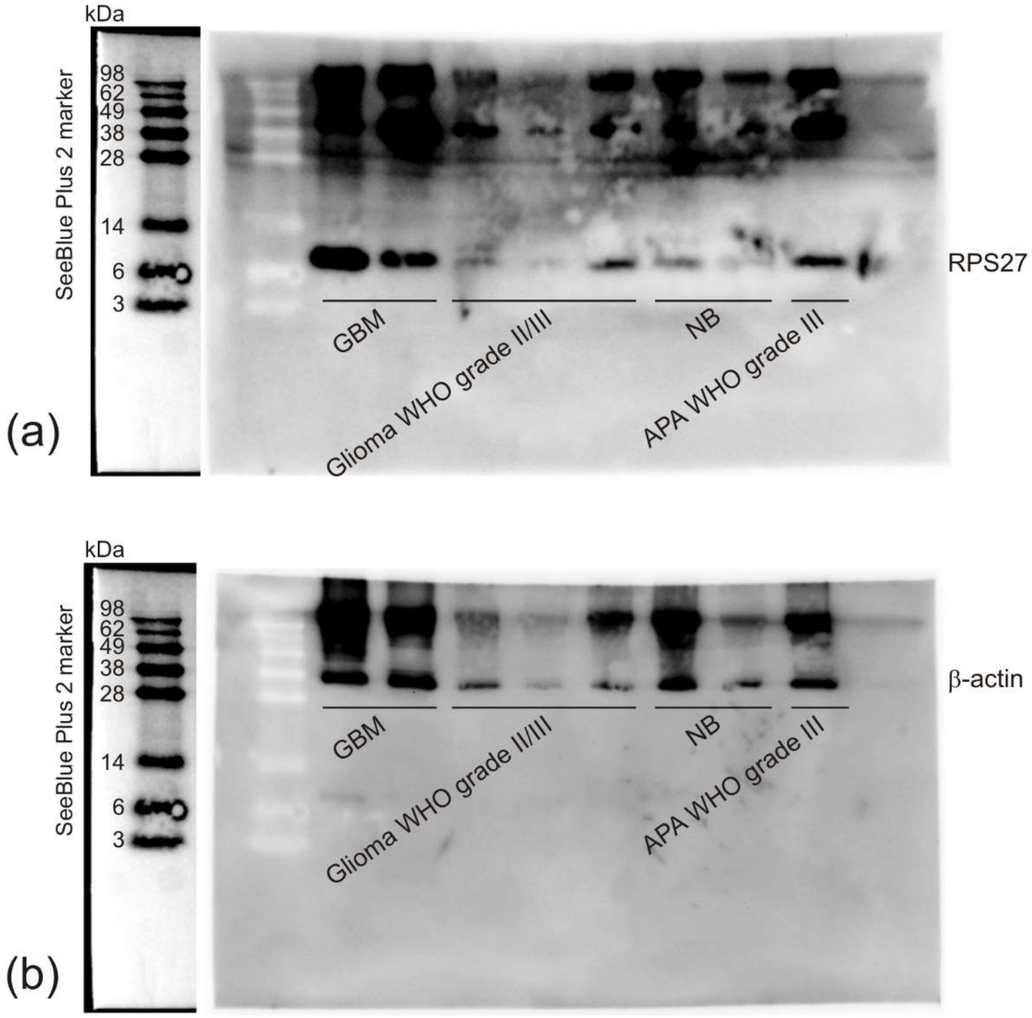

Figure S3. Unaltered Western blots of RPS27 and β-actin protein expression in randomly selected glioma samples. (**a**) Detection of RPS27 and (**b**) of the housekeeping protein β-actin as a loading control on 15% SDS gels. 100 µg protein was loaded in both cases.

Table S1. Average optical density (OD) of DAB-stained NB and tumor specimens analyzed in Figure 5.

Tissue type	Average OD	SD
NB grey matter	0.378	0.099
NB white matter	0.302	0.036
Gliomas WHO grade II/III	0.296	0.100
GBM	0.294	0.093

OD = optical density; SD = standard deviation; NB = normal brain tissue; WHO = world health organization; GBM = glioblastoma.

Table S2. Antibodies used for immunohistochemistry and double-fluorescence staining.

Antibody	Target	Marker	Species	Dilution	Method	Manufacturer
Mouse Anti-Neuronal Nuclei Monoclonal Antibody	NeuN	Neuronal marker	Mouse	1:500	DAB, Flourescence	Millipore (Burlington, USA)
Anti-IDH1 R132H/DIA-H09 Mouse monoclonal anti-brain tumor marker	IDH1 R132H	IDH1 R132 mutation status	Mouse	1:20	DAB, Fluorescence	Dianova (Hamburg, Germany)
Ki67 Antibody M7240	Ki67	Proliferation marker	Mouse	1:800	DAB, Fluorescence	Dako (Glostrup, Denmark)
CD68 (Macrophage Marker) Ab-4 (Clone PG-M1)	CD68	Macrophage marker	Mouse	1:200	DAB, Fluorescence	Dianova (Hamburg, Germany)
GFAP Monoclonal Antibody (GA5)	GFAP	Astrocytic marker	Mouse	1:100	DAB, Fluorescence	Invitrogen (Waltham, USA)
Mouse Monoclonal to MPS1	RPS27	RPS27 protein expression	Mouse	1:200	DAB	ProMab Biotechnologies (Richmond, USA)
MPS1 Polyclonal Antibody	RPS27	RPS27 protein expression	Rabbit	1:500<br>1:400	Fluorescence<br>Western-blot	ThermoFisher Scientific (Waltham, USA)
Monoclonal Anti-β-Actin-Peroxidase Antibody	β-actin	Housekeeping gene	Mouse	1:2500	Western-blot	Sigma-Aldrich (St. Louis, USA)
Goat anti-Rabbit IgG (H+L), Highly Cross-Adsorbed Secondary Antibody, Alexa Fluor Plus 555	Secondary antibody versus rabbit	Secondary antibody	Goat	1:400	Fluorescence	ThermoFisher Scientific (Waltham, USA)
Goat anti-Mouse IgG (H+L), Highly Cross-Adsorbed Secondary Antibody, Alexa Fluor Plus 488	Secondary antibody versus mouse	Secondary antibody	Goat	1:400	Fluorescence	ThermoFisher Scientific (Waltham, USA)

Table S3. Macro used for semi-automatic picture evaluation.

Macro commands

```
input = getDirectory("Input directory");
output = getDirectory("Output directory");
Dialog.create("File type");
Dialog.addString("File suffix: ", ".tif", 5);
Dialog.show();
suffix = Dialog.getString();
processFolder(input);
function processFolder(input) {
  list = getFileList(input);
  for (i = 0; i < list.length; i++) {
        if(File.isDirectory(input + list[i]))
              processFolder("" + input + list[i]);
        if(endsWith(list[i], suffix))
              processFile(input, output, list[i]);
  }
}
function processFile(input, output, file) {
open(input + File.separator + file);
name=getTitle();
run("Colour Deconvolution", "vectors=[H DAB]");
selectWindow (name+"-(Colour_1)");
run("Conversions...", "scale");
run("8-bit");
un("Subtract Background...", "rolling=500 light");
run("Gaussian Blur...", "sigma=5");
un("Threshold...");
selectWindow (name+"-(Colour_1)");
waitForUser("Set the threshold and press OK, or cancel to exit macro. DO NOT press apply
in Threshold window.");
run("Convert to Mask");
selectWindow (name+"-(Colour_1)");
run("Watershed");
run("Analyze Particles...", "size=400-2000 summarize");
selectWindow (name+"-(Colour_2)");
run("Measure");
run("Conversions...", "scale");
run("8-bit");
run("Subtract Background...", "rolling=500 light");
run("Gaussian Blur...", "sigma=5");
run("Threshold...");
selectWindow (name+"-(Colour_2)");
```

```
    waitForUser("Set the threshold and press OK, or cancel to exit macro. DO NOT press apply in Threshold window.");
    run("Convert to Mask");
    selectWindow (name+"-(Colour_2)");
    run("Watershed");
    run("Analyze Particles...", "size=1000-3000 summarize");
    run("Close All");
}
```

© 2020 by the authors. Licensee MDPI, Basel, Switzerland. This article is an open access article distributed under the terms and conditions of the Creative Commons Attribution (CC BY) license (http://creativecommons.org/licenses/by/4.0/).
